# *In Vivo* Evolution of Bacterial Resistance in Two Cases of *Enterobacter aerogenes* Infections during Treatment with Imipenem

**DOI:** 10.1371/journal.pone.0138828

**Published:** 2015-09-23

**Authors:** Nadège Philippe, Laure Maigre, Sébastien Santini, Elizabeth Pinet, Jean-Michel Claverie, Anne-Véronique Davin-Régli, Jean-Marie Pagès, Muriel Masi

**Affiliations:** 1 UMR-MD1 –Transporteurs Membranaires, Chimiorésistance et Drug Design–Aix-Marseille Université et Institut de Recherche Biomédicale des Armées (IRBA), 27 Boulevard Jean Moulin, 13385 Marseille cedex 05, France; 2 Laboratoire Information Génomique et Structurale–CNRS UMR7256 –Institut de Microbiologie de la Méditerranée (IMM, FR3479) Parc Scientifique de Luminy– 163 Avenue de Luminy–Case 934 FR-13288, Marseille cedex 09, France; Arizona State University, UNITED STATES

## Abstract

Infections caused by multidrug resistant (MDR) bacteria are a major concern worldwide. Changes in membrane permeability, including decreased influx and/or increased efflux of antibiotics, are known as key contributors of bacterial MDR. Therefore, it is of critical importance to understand molecular mechanisms that link membrane permeability to MDR in order to design new antimicrobial strategies. In this work, we describe genotype-phenotype correlations in *Enterobacter aerogenes*, a clinically problematic and antibiotic resistant bacterium. To do this, series of clinical isolates have been periodically collected from two patients during chemotherapy with imipenem. The isolates exhibited different levels of resistance towards multiple classes of antibiotics, consistently with the presence or the absence of porins and efflux pumps. Transport assays were used to characterize membrane permeability defects. Simultaneous genome-wide analysis allowed the identification of putative mutations responsible for MDR. The genome of the imipenem-susceptible isolate G7 was sequenced to closure and used as a reference for comparative genomics. This approach uncovered several *loci* that were specifically mutated in MDR isolates and whose products are known to control membrane permeability. These were *omp35* and *omp36*, encoding the two major porins; *rob*, encoding a global AraC-type transcriptional activator; *cpxA, phoQ* and *pmrB*, encoding sensor kinases of the CpxRA, PhoPQ and PmrAB two-component regulatory systems, respectively. This report provides a comprehensive analysis of membrane alterations relative to mutational steps in the evolution of MDR of a recognized nosocomial pathogen.

## Introduction

Multidrug resistance (MDR) is a significant problem for treatment of bacterial infections worldwide. Although carbapenems are the last β-lactams that retain a nearly-universal activity against Gram-negative bacteria, carbapenem-resistant *Enterobacteriaceae* (CRE) have emerged as important cause of morbidity and mortality among hospital-acquired and long-term care-associated infections. A 2013 report on antibiotic resistance threats by the American Centers for Disease Control and Prevention (CDC) estimated that 730,000 infections and 3,400 deaths were caused annually by CRE in the US alone [1]. This report also classified CRE as an urgent threat. Therefore, it is of critical importance to understand the molecular mechanisms underlying the emergence of MDR and to develop therapeutic alternatives for these problematic bacteria.

*Enterobacter aerogene*s is one of the most common CRE together with *Klebsiella pneumoniae* and *Escherichia coli*. Since the early 1990s, *E*. *aerogene*s has emerged as an important MDR pathogen, responsible for nosocomial infections, including respiratory and urinary tracts infections, bacteremia, sepsis and post-chirurgical infections [2–4]. The emergence of MDR strains of *E*. *aerogenes* is closely related to the clinical use of broad-spectrum β-lactams and carbapenems [5–10]. Several mechanisms account for MDR, including carbapenem resistance, in this species. Resistance to β-lactams is mainly due to enzymatic degradation by a chromosomal cephalosporinase, plasmid-mediated broad spectrum β-lactamases, and recently acquired carbapenemases [4, 6, 7, 9–14]. Changes in the bacterial cell envelope are also a key contributor to the high levels of resistance towards β-lactams, including carbapenems, and other antibiotic families such as fluoroquinolones [6–10, 12, 15–18]. These changes include quantitative or qualitative modifications of outer membrane porins [6–10, 16, 18–20], and increased levels of efflux pumps [12, 17, 18, 21, 22]. In *Enterobacteriaceae*, MDR is controlled by several regulatory genes and external factors [15]. MarA, RamA and SoxS are among the best studied MDR regulators. Several mutations have been identified in *marRAB*, *ramAR* or *soxRS* in clinical isolates and associated to MDR [15]. These master regulatory pathways are interconnected and function by up-regulating the expression efflux proteins and down-regulating the expression of porins. In addition, MDR can be induced by clinically used antibiotics such as imipenem. This phenomenon has been observed *in vitro*, when bacteria are trained to grow in the presence of increasing concentrations of drugs and, *in vivo*, during antibiotic treatment of infected patients [6–10, 21, 23]. Imipenem and others effectors probably act by activating MDR regulators such as MarA or RamA.

So far, the vast majority of studies on clinical isolates were aimed to identify mutations in the currently known resistance-conferring targets (i.e. promoter or structural mutations in porin genes and mutations in local or global MDR regulatory genes) [24–28]. This approach is biased in that some MDR isolates have no detectable alterations in any of these targets, suggesting that other *loci* are involved in MDR that have yet to be identified. In addition, clinical isolates are often collected from multiple patients after a local outbreak or obtained from human infections at diverse geographic locations. This approach does not allow the identification of steps of the *in vivo* evolution of drug resistance. We have previously reported the emergence of MDR in *E*. *aerogenes* isolates recovered from several patients during their clinical course in French hospitals [7]. All isolates were isogenic as indicated by randomly amplified polymorphic DNA (RAPD) genotyping and belonged to a prevalent epidemiologic clone that contains a plasmid encoding a TEM-24 extended-spectrum β-lactamase (ESBL). Treatments with imipenem lead to MDR phenotypes associated to outer membrane permeability defects. Yet, virtually nothing was known about how these resistance traits were acquired. In this work, we selected series of clinical isolates obtained from two patients infected with *E*. *aerogenes*. Five isolates from patient P and seven isolates from patient G, covering the entire treatment period, were phenotypically characterized and submitted to whole-genome sequencing. This approach uncovered genetic determinants of resistance in *E*. *aerogenes*, which are all involved in outer membrane permeability control. These results led to the identification of promising targets for the development of drugs that would function as chemosensitizers to potentiate uptake and activity of existing antibiotics.

## Materials and Methods

### *E*. *aerogenes* strains, growth media, antibiotics and chemicals

The two series of *E*. *aerogenes* isolates came from two patients hospitalized in 1997 and were obtained from two different healthcare facilities in the south of France [7] (see S1 Table for sampling sites). Patient P was treated with imipenem and four isolates have been recovered (P1 to P4). Patient G was treated with imipenem and cefpirome, and 7 isolates have been recovered (G1 to G7). All strains were primarily isolated on Mueller Hinton (MH) agar. Because P4 constantly gave rise to heterogeneous colonies after overnight growth at 37°C, we decided to include both small and big colonies in our study. These will be hereafter referred to as P4 and P5, respectively. We selected G7 as a representative *E*. *aerogenes* isolate for *de novo* sequencing and comparative genome analysis based upon its susceptibility to imipenem (see S1 Table for detailed susceptibility profiles). *E*. *aerogenes* ATCC13048 (KCTC2190) [29] and ATCC15038 are from our laboratory stock and were used as antibiotic susceptible strains. Unless indicated otherwise, all strains were routinely cultured in Luria Bertani (LB) broth at 37°C with aeration. All media were purchased from Difco Laboratories. Antibiotics and chemicals are from Sigma.

### Determination of minimal inhibitory concentrations

Minimal inhibitory concentrations (MICs) were determined by broth dilution method in 96-wells microplates (Greiner Bio-One). Bacteria were cultured to exponential phase in cation adjusted MH II broth. Approximately 2×10^5^ colony forming units (CFU) were inoculated into 200 μL of fresh medium containing two-fold serial dilutions of each antibiotic in the absence or in the presence of additive compounds: polymyxin B nonapeptide (PMBN), phenylalanine arginine β-naphthylamide (PAβN), 1-(1-Naphtyl Methyl)-piperazine (NMP), EDTA, or clavulanic acid. PMBN was used as a membrane permeabilizer at a final concentration of 102.4 μg/ml. PAβN and NMP were used as efflux inhibitors at a final concentration of 20 μg/ml and 100 μg/ml, respectively. EDTA was added at a final concentration of 0.5 mM and used as a chelator of divalent cations to disturb the stability of lipopolysaccharide (LPS). Clavulanic acid was added at a final concentration of 4 μg/ml and used as a β-lactamase inhibitor. After an incubation of 18 h at 37°C, bacterial growth was evaluated by using 2-(4-iodophenyl)-3-(4-nitrophenyl)-5-phenyl-2H-tetrazolium chloride as a viability dye. MICs were determined as the lowest concentration of each antibiotic that prevented growth. Values are means of at least three independent experiments and are expressed in μg/ml. Isolates were classified as susceptible (S), intermediate (I) or resistant (R) to the antibiotics tested according to the recommendations of the Antibiogram Committee of the French Society for Microbiology (http://www.sfm-microbiologie.org).

### Hoechst 33342 influx assays

Hoechst 33342 (H33342) was used as a fluorescent probe for permeability of the outer membrane bilayer. All strains were cultured LB broth until they reached an OD_600_ between 1.8 and 2.4. Cells were harvested by centrifugation at room temperature, washed once and resuspended in potassium phosphate buffer (PPB) (20 mM K_2_HPO_4_, pH 7.0) in the presence of 50 μM carbonyl cyanide *m*-chlorophenylhydrazone (CCCP) to an OD_600_ of 0.4. 100 μl of bacterial suspensions were transferred in black flat bottom 96 well half area microplates (Greiner Bio-One). H33342 was added to the mixtures at a final concentration of 5 μM and the fluorescence of the H33342 bound to nucleic acids generated by the influx of H33342 into the cells was determined at room temperature by using an Infinite® M200 Pro (TECAN) spectrofluorometer with excitation and emission wavelengths of 360 and 500 nm, respectively. CCCP was used to collapse the protonmotive force (pmf) across the inner membrane. Slopes resulting from the initial increase in H33342 fluorescence were calculated and expressed as changes in fluorescence intensity per second (ΔFI/s). Total accumulation levels were calculated as areas under the curves. All values are means of at least three independent experiments.

### Fleroxacin accumulation

All strains were cultured overnight in LB broth until they reached an OD_600_ of 0.6. Cells were harvested by centrifugation, washed once and concentrated 10-fold in 50 mM NaPi pH 7.0 supplemented with 5 mM MgCl_2_ to approximately 10^10^ CFU/ml. Zero-time samples were removed and fleroxacin was added to a final concentration of 64 μg/ml in the absence or in the presence of 10 μM of CCCP. An incubation of 30 min at 37°C was chosen based on preliminary kinetic experiments to reach saturation (data not shown). 0.8 ml aliquots were then removed, loaded onto a 1.1 ml cushion of 1 M sucrose and immediately centrifuged for 5 min at 4°C. The pellets were resuspended in 0.5 ml of 0.1 M Glycin-HCl pH 3.0 and incubated for 2 h at room temperature to lyse the cells. The lysates were centrifuged for 15 min at 4°C to remove any cell debris and 100 μl-aliquots of the supernatants were transferred in black flat bottom 96 well half area microplates. Spectra were recorded by using an Infinite® M200 Pro (TECAN) spectrofluorometer with excitation and emission wavelengths of 290 nm and 310–700 nm, respectively. The concentration of fleroxacin in the supernatants was estimated after normalization of the spectra to their corresponding tryptophan emission peak at 356 nm. The antibiotic-free control was subtracted from the values obtained for each assay. All values are means of at least three independent experiments.

### 1,2’-dinaphthylamine efflux assays

The procedure for real-time efflux assays of 1,2’-dinaphthylamine (1,2’-DNA) was adapted from Bohnert *et al*. [30]. Briefly, all strains were cultured overnight in MH II at 37°C. Cells were harvested by centrifugation, washed once and resuspended in PPB supplemented with 1 mM MgCl_2_ (Mg-PPB) to a final OD_600_ of 0.25. Cells were first deenergized by an overnight incubation in Mg-PPB with 5 μM CCCP and loaded with 32 μM 1,2’-DNA at 37°C in the dark. Cells were then centrifuged, washed, resuspended in Mg-PPB and 100 μl of bacterial suspensions were transferred in black flat bottom 96 well half area microplates. Fluorescence was followed over 60 sec and 1,2’-DNA efflux was triggered after rapid reenergization with 5 μl of 1 M glucose and monitored for another 250 sec. All measurements were performed at 37°C by using an Infinite® M200 Pro (TECAN) spectrofluorometer with excitation and emission wavelengths of 370 nm and 420 nm, respectively. Slopes resulting from the initial decrease in 1,2’ DNA fluorescence were calculated and expressed as changes in fluorescence intensity per second (ΔFI/s). Values are means of at least three independent experiments.

### Protein methods

All strains were cultured overnight at 37°C in MH broth. Aliquots of cells were harvested by centrifugation and resuspended in Laemmli sample buffer to an OD_600_ of 2.0. After 5 min at 95°C, protein samples (10 μl ∼ 0.2 OD units) were run on polyacrylamide 10% mini gels and transferred onto nitrocellulose membranes. Membranes were probed with primary antibodies raised against *Escherichia coli* OmpF (1:5,000), OmpC (1:5,000), AcrA (1:10,000), AcrB (1:2,000) or TolC (1:2,000). Goat anti-rabbit horseradish-peroxydase conjugated immunoglobulin G secondary antibodies and a chemiluminescent kit (BioRad) were used for detection.

Outer membrane porins were identified and characterized by mass spectrometry. Briefly, strains were cultured overnight in osmotically-controlled media—Nutrient broth (NB, 46(±3) mOsmol/kg) and NB supplemented with 20% sorbitol (NBS, 1290(±23) mOsmol/kg)—, in which the porin ratio is tightly regulated. Cell pellets from 100 ml cultures were washed once and resuspended in 3 ml lysis buffer containing 20 mM Tris-HCl, pH 7.5 and 1 mM MgCl_2_. Bacterial cells were broken by one passage through cell disrupter at 2 kBars. Unlysed cells were removed by centrifugation (8,000 × g for 20 min) and supernatant was ultracentrifuged at 105,000 × g for 1 h. Pellets containing whole cell-envelope were mixed with Laemmli sample buffer, heated for 5 min at 95°C, and membrane proteins were separated by SDS-PAGE. Urea (4 M) was added to the SDS-polyacrylamide running gel in order to better resolve Omp35 and Omp36. Bands of interest were excised, digested with trypsin, and analyzed by Nano-LC MS/MS at the SICaPs (Imagif, Service d’identification et de caractérisation des protéines par spectrométrie de masse, Plateforme SICaPS, Campus CNRS, Gif-sur-Yvette).

### *E*. *aerogenes* G7 *de novo* DNA sequencing and assembly

Total DNA of *E*. *aerogenes* G7 was isolated from an overnight culture using the Wizard® Genomic DNA Purification Kit (Promega) according to the manufacturer’s instructions, except for the following modifications. During the protein precipitation step, the samples were incubated on ice for 10 min and centrifuged for 10 min at 4°C. The supernatants were recovered and centrifuged again to carefully eliminate any precipitates and to ensure an optimal DNA quality for sequencing. Total DNA was qualitatively and quantitatively checked by migration on agarose gels and by using a NanoDrop™ spectrophotometer. The genome of G7 was sequenced *de novo* on PacBio RS, assembled and annotated by GATC Biotech AG (Cologne, Germany). Genome annotation was performed with Glimmer 3 [31] and RAST (http://rast.nmpdr.org/) [32, 33]. The genome of EAG7 has been deposited in GenBank under National Center for Biotechnology Information (NCBI) accession numbers CP011539 (EAG7 chromosome) and CP011540 (EAG7 plasmid pGPN1).

### Genome sequencing of the *E*. *aerogenes* isolates and detection of variations

Total DNA was isolated from overnight cultures of the twelve *E*. *aerogenes* isolates, including G7, as described above. Genomes were sequenced by using an Illumina HiSeq 2000 sequencer (100 bp paired end) and HiSeq reads were mapped to the G7 assembled genome by GATC using BWA [34] with the default parameters. SNPs and InDels were called using GATK's UnifiedGenotyper. All SNPs and InDels were individually checked *in silico* using the Tablet software [35], to eliminate GATK’s false positives. SNPs and InDels that lead to modifications of predicted coding sequences and that were specifically present in resistant isolates were confirmed by PCR and sequencing (Eurofins, MWG, Ebersberg, Germany). HiSeq reads that could not be mapped to the PacBio assembled genome of G7 were considered as regions of divergence. Genome assembly from the HiSeq data was first performed by using Velvet 1.2.10 [36] and resulted in several contigs for each isolate. The contigs of the twelve genomes were then aligned relative to the assembled genome of G7 and compared with progressiveMauve [37]. Specific sequences from each strain were isolated and analyzed by blastn [38]. Phage-related sequences were searched by using PHAST (http://phast.wishartlab.com) [39]. Insertion sequences (IS) were characterized by using the ISFinder database (http://www-is.biotoul.fr) [40]. Putative integrative conjugative elements (ICEs) and integrative mobilizable element (IMEs) were found by performing iterative similarity searches followed by protein clustering. With this approach, ICEs would correspond to recombinase/integrase-bounded regions containing type IV secretion system (T4SS) proteins and MobA-type relaxase(s), while IMEs would comprise relaxase(s) with no neighbor T4SS. Metadata were deposited in the NCBI Sequence Read Archive under the accession SRP056584.

## Results

### Clinical isolates and evolution of antibiotic resistance

Patient P was hospitalized without any sign of infection. However, the patient was immunocompromised and rapidly developed a respiratory tract infection associated to *E*. *aerogenes*. Isolate P1 corresponded to the first isolation of *E*. *aerogenes* from a bronchial aspirate and was susceptible to almost all tested antibiotics including gentamicin (data not shown), carbapenems, third (ceftazidime and cefotaxime) and fourth (cefepime) generation cephalosporins, and quinolones (Table 1 and S1 Table for detailed MIC values). From that point, patient P was treated with imipenem. Four days after the first use of imipenem, P2 was isolated from a new bronchial aspirate. Although this strain still retained susceptibility to gentamicin, it was fully resistant to quinolones, carbapenems and cephalosporins. The last strains P4 and P5, isolated after twelve days of treatment, presented the same MDR phenotype. Interestingly, the mid-term isolate P3 was resistant to quinolones but susceptible to carbapenems, suggesting the co-existence of two subpopulations at day 4.

**Table 1:** Phenotypic characterization of *E*. *aerogenes* strains.

	Patient P															Patient G												
Nb of days	1	2	3	4	5	6	7	8	9	10	11	12		13	14	7	14	21	28	35	42	49	56	63	70		91	
Imipenem treatment			+	+	+	+	+	+	+	+	+	+	+	+	+	+	+	+	+	+			+	+	+			
Cefpirome treatment																							+					
Sampling site^*a*^	T						T	T				T	T			U				V	S	V			V	B	T	
Chloramphenicol	R						R	R				R	R			R				R	R	R			R	R	R	S
Fleroxacin	S						R	R				R	R			R				R	R	R			R	R	R	S
Norfloxacin	S						R	R				R	R			R				R	R	R			R	R	R	S
Nalidixic acid	S						R	R				R	R			R				R	R	R			R	R	R	S
Erythromycin	R						R	R				R	R			R				R	R	R			R	R	R	R
Imipenem	S						R	S				R	R			S				R	S	S			R	R	S	S
Ertapenem	S						R	S				R	R			S				R	S	S			R	R	S	S
Cefepime	S						R	S				R	R			R				R	S	S			R	R	S	S
Ceftazidime	S						R	R				R	R			R				R	R	R			R	R	R	S
Cefotaxime	S						R	R				R	R			R				R	S	S			R	R	S	S
Polymyxin B	S						S	S				S	S			S				S	S	S			R	S	S	S
Tetracycline	S						R	R				R	R			S				R	R	R			R	R	R	S
Isolate	P1						P2	P3				P4	P5			G1				G2	G3	G4			G5	G6	G7	ATCC15038
Porins^*b*^ ^*^	+						-	+				-	-			+				-	+	+			-	-	+	+
Efflux Pump Proteins^*b*^	+						+	+				+	+			+				+	+	+			+	+	+	+

*E*. *aerogenes* isolates were recovered periodically from two patients undergoing chemotherapy with imipenem—P1 to P5 from patient P and G1 to G7 from patient G. ATCC15038 was used as a reference strain. MICs were determined by broth microdilution in MHII broth as described in Materials and Methods. For clarity, strains have been classified as R for resistant and S for susceptible. Detailed MICs are presented in supplementary information (S1 Table). Different classes of antibiotics were tested: phenicols (chloramphenicol), quinolones (nalidixic acid, fleroxacin and norfloxacin), macrolides (erythromycin), carbapenems (imipenem and ertapenem), cephalosporins (cefepime, ceftazidime and cefotaxime), tetracycline and polymyxin B. Gentamicin is often the last option to treat MDR strains. For this reasons, we chose not to include this antibiotic in our assays but we refer to MIC values obtained from the healthcare facilities.

^*a*^ Sampling sites: U, urine; V, vagina; S, Sputum; B, blood; T, trachea.

^*b*^ The presence of porins and efflux pump proteins were determined immunoblots using specific antibodies directed against *Escherichia coli* OmpF, OmpC, AcrA, AcrB or TolC.

*Mass spectrometry analysis indicated that porin-plus isolates only express Omp36 whereas strain ATCC15038 expresses both Omp36 and Omp35 (S1 Fig and S2 Table).

Patient G was hospitalized in an intensive care unit in a different geographic zone. The first strain of *E*. *aerogenes* was isolated from the urinary tract three months after hospitalization. Isolate G1 was susceptible to gentamicin, carbapenems and cephalosporins but resistant to fluoroquinolones. After four weeks of treatment with imipenem, *E*. *aerogenes* disappeared from the urinary tract, but reappeared as a MDR strain in the vaginal tract (isolate G2). From that point, treatment was stopped for one month. During this period, isolates G3 and G4 were cultured from sputum and vagina, respectively, and showed a reversion of resistance to carbapenems. Patient G finally received cefpirome for a few days and imipenem for two weeks. Isolates G5 and G6 were cultured from vagina and blood at the end of this period, and both showed similar MDR phenotypes. Three weeks after imipenem treatment was stopped, isolate G7 was cultured from a bronchial aspirate, with a new reversion of susceptibility to carbapenems.

### Membrane permeability and efflux activity in the *E*. *aerogenes* isolates

Outer membrane porins represent the unique specific route of the uptake of β-lactam antibiotics [16]. Therefore, porin expression levels in the isolates were analyzed by western-blot using antibodies directed against denatured *E*. *coli* OmpF and OmpC (data not shown). Reference strains ATCC13048 and ATCC15038 were included as controls, as the former expresses only Omp36 (OmpC) and the latter expresses both Omp36 and Omp35 (OmpF). One cross-reacting band was detected in carbapenem-susceptible strains only. This band was identified as Omp36 by mass spectrometry (S1 Fig and S2 Table).

**Fig 1:**
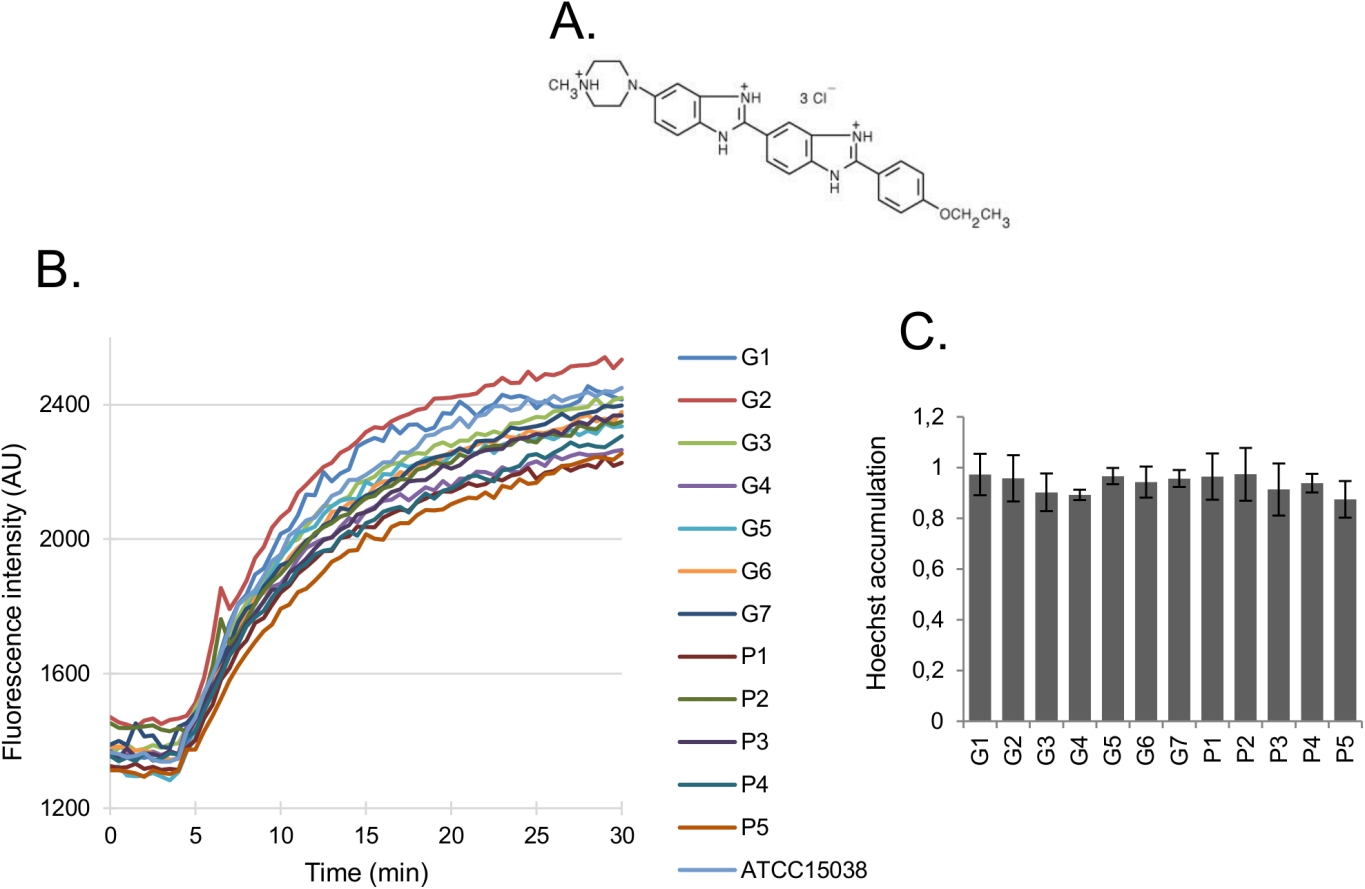
Hoechst 33342 influx into *E*. *aerogenes* strains. Strains were grown in LB broth and cells were harvested at the beginning of the stationary phase. Cells were washed, resuspended in PPB pH 7.0, and the H33342 influx was assayed in the presence of 50 μM CCCP. A. Chemical structure of H33342. B. Kinetics of H33342 influx. AU, arbitrary units. C. Total accumulation in *E*. *aerogenes* isolates after normalization to that of strain ATCC15038. All values are means of at least three independent experiments.

To examine outer membrane permeability, intact bacterial cells were incubated with H33342. Once H33342 crosses the outer membrane, it diffuses through the inner membrane very rapidly and binds to nucleic acids in the cytoplasm, producing a fluorescent signal. Thus, one can determine the rate-limiting step (i.e. the influx across the outer membrane) from the rate of fluorescence increase. H33342 is a large (> 12 Å across), hydrophobic dye, with delocalized positive charges, thus unlikely able to diffuse through the narrow porin channel. Resuspension of cells in divalent cation-free buffer was expected to destabilize the LPS leaflet of the outer membrane and active efflux was blocked by the presence of the pmf inhibitor CCCP. When H33342 total accumulation levels and entry rates were compared, all strains showed similar permeability, although slightly lower to that of the reference strain ATCC15038 (Fig 1 and S3 Table). This result suggests the absence of major remodeling processes that would account for outer membrane impermeability in the isolates. Nevertheless, it is important to note that this assay does not reflect porin alterations.

**Fig 2:**
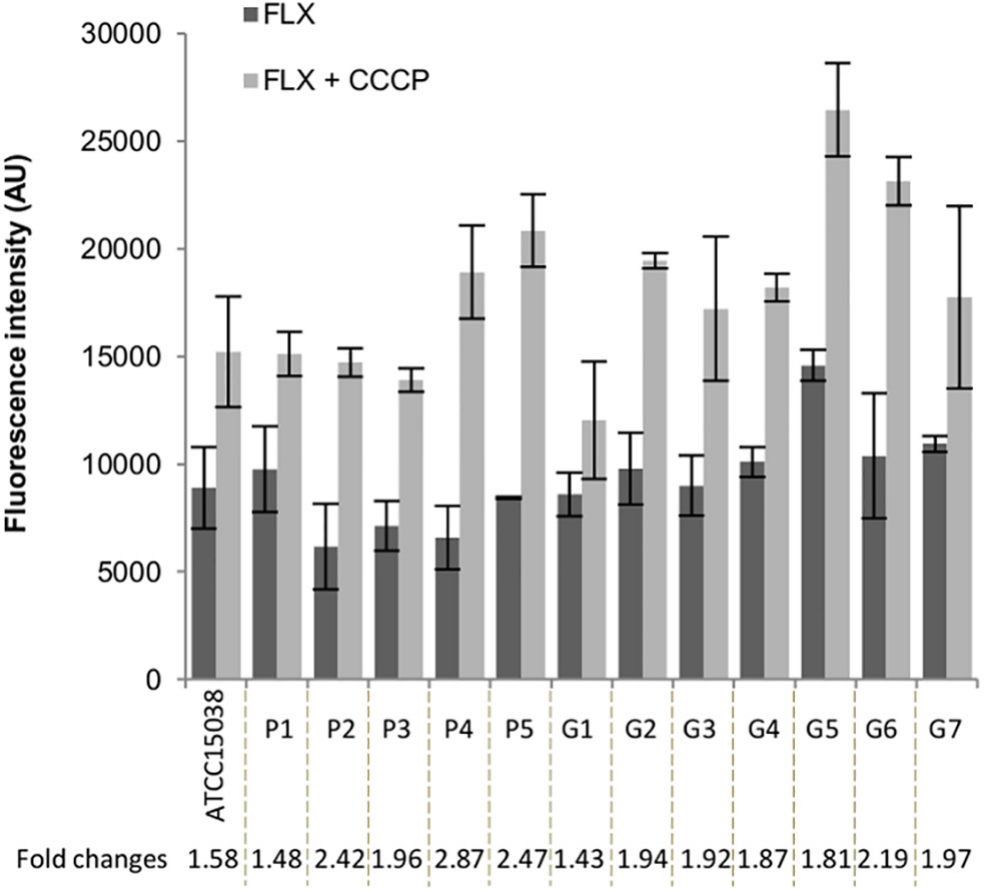
Fleroxacin accumulation in *E*. *aerogenes* strains. Strains were grown in LB broth and cells were harvested at mid-log phase. Cells were washed and concentrated 10-fold in NaPi pH 7.0 supplemented with Mg^2+^. Fleroxacin (FLX) was added to the bacterial suspensions in the absence or in the presence of 10 μM CCCP. After a 30 min incubation time, bacterial lysates were prepared as described in Materials and Methods. The concentration of FLX in the lysates was estimated after normalization of the spectra to their corresponding tryptophan emission peak at 356 nm. The antibiotic-free control time-zero was subtracted from the values obtained for each assay. All values are means of at least three independent experiments.

A common characteristic of the MDR isolates of *E*. *aerogenes* is their high level of resistance towards quinolones (Table 1 and S1 Table). Hence, we used a fluorometric assay based on determination of the time course accumulation of a fluoroquinolone inside the cells to assess efflux [41]. Fig 2 shows the intracellular accumulation of fleroxacin in the *E*. *aerogenes* isolates. Higher accumulation was observed in the presence of CCCP, confirming the presence of an active efflux in all the strains. Of note, MDR isolates from patient P—P2, P4 and P5—showed a more than 2-fold accumulation of fleroxacin in the presence of CCCP (Fig 2). However, accumulation levels in the different isolates could not be related to the presence or the absence of outer membrane porins. This could be explained by the fact that membrane permeability plays only a secondary role in quinolone uptake, in sharp contrast to other antibiotics such as β-lactams.

**Fig 3:**
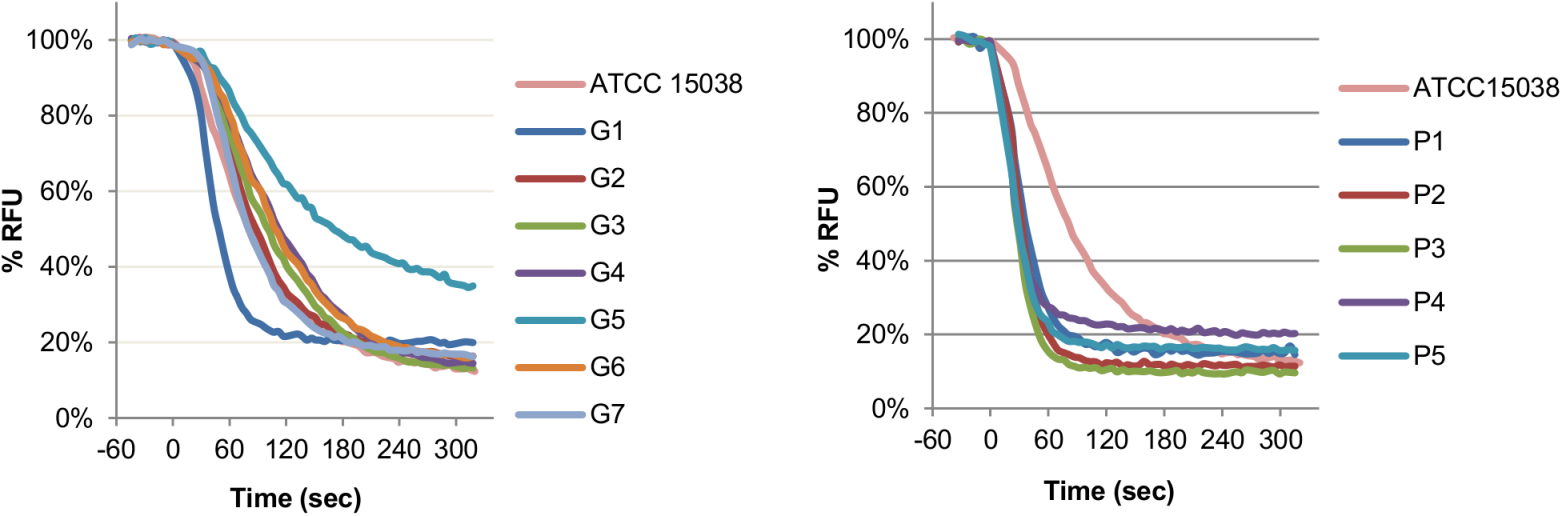
Real-time efflux of 1,2'-DNA in *E*. *aerogenes* strains. Strains were grown in LB broth for 24 h at 37°C and cells were harvested by centrifugation. Cells were deenergized by an overnight incubation in Mg-PPB pH 7.0 with 5 μM CCCP and loaded with 1,2’-DNA. Cells were then washed, resuspended in Mg-PPB pH 7.0 and 1,2’-DNA efflux was monitored after rapid reenergization with glucose. RFU, relative fluorescence units. All values are means of at least three independent experiments.

We also used a real-time efflux assay with cells that have been preloaded with the fluorescent dye 1,2'-DNA. Consistently with previous results, isolates from patient P display higher efflux rates in comparison to ATCC15038 and isolates from patient G (with the exceptions of G1 and G5) (Fig 3 and S3 Table).

### Genomic features of *E*. *aerogenes* G7 and others clinical isolates

In order to identify putative mutations that could account for porin regulation all twelve *E*. *aerogenes* isolates were submitted to whole-genome sequencing and mapped to G7, selected as a representative *E*. *aerogenes* isolate, as describes in Materials and Methods. The average depth of coverage for the twelve isolates was 388-fold, and over 95% of the newly sequenced and assembled *E*. *aerogenes* G7 genome was covered after mapping (Table 2).

**Table 2:** Summary of genome analysis.

Isolate		P2-P5	G1	G2-G6
Coverage (% to the reference strain after mapping)	Chromosome pGPN1	98.2 95.1	96.6 95.2	99.1 95.3
Reads (∼ % of mapped)		85	92	93
SNPs and InDels		70–72	92	7–11

**Fig 4:**
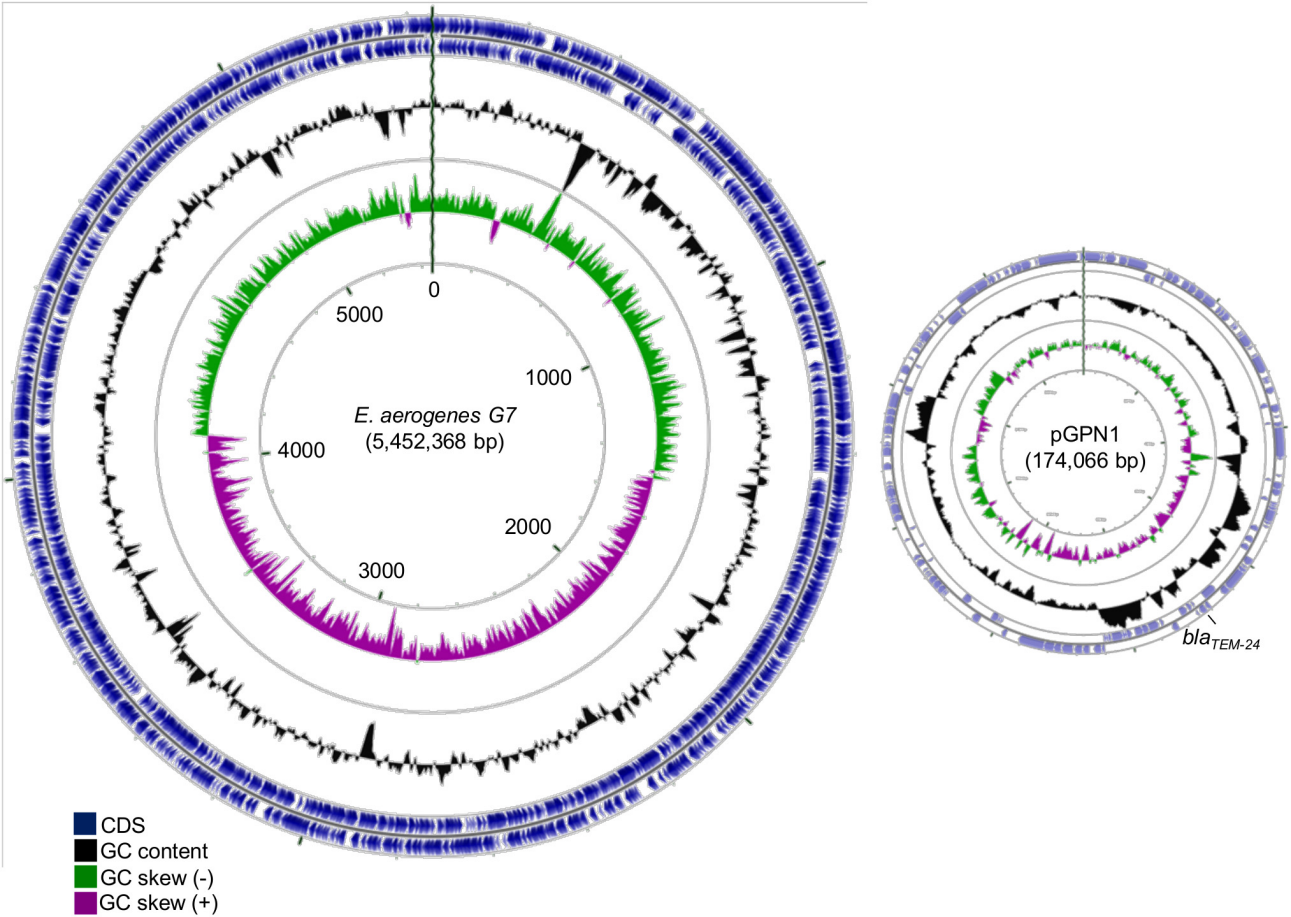
Genome of *E*. *aerogenes* G7. This figure was generated with GCviewer (http://stothard.afns.ualberta.ca/cgview_server/) and shows the map of the chromosome and plasmid pGPN1 (174 Kbp) of *E*. *aerogenes* isolate G7 obtained by *de novo* sequencing and assembly.

The genome of *E*. *aerogenes* G7 (EAG7) includes a chromosome of 5,452,368 bp with a GC content of 54.92% and 2 plasmids: a large plasmid of 174,066 bp (pGPN1) and a smaller plasmid of 25,284 bp (pGPN2), from which we only obtained a partial sequence of about 9 Kbp but was likely identical to pNJS258C2 found in a carbapenem-resistant clinical isolate of *K*. *pneumoniae* [42] (Fig 4 and S1 Dataset). These two plasmids were present in all the sequenced strains and contained multiple antibiotic resistance markers, including *bla*_*TEM-24*_ on pGPN1 (see S3 Dataset for a complete list). Isolates P2 to P5 have a third plasmid of 34,826 bp similar to pN-Cit found in *Citrobacter freundii* strain CFSTE [43]. The functional annotation of the chromosome indicates a total of 5,267 predicted coding sequences (CDSs) (Table 3). The G+C content of the EAG7 chromosome is 54.92%, which is in agreement with values previously reported for KCTC2190 (54.8%) [29] and EA1509E (55%) [44]. There were 8 ribosomal operons and 88 transfer RNAs (tRNAs) identified in EAG7, with 87 specific for the biosynthesis of all the 20 standard amino acids and 1 for the selenomethionine biosynthesis, which was identical to those of EA1509E but different from KCTC2190 that carries a total of 109 structural RNAs (Table 3 and S1 Dataset). Regarding the chromosome size, EAG7 resembles the “killer bug” EA1509E, while the strain KCTC2190 presents a smaller chromosome size. This is probably due to the accumulation of genes related to their host-associated life-style and adaptation under anti-microbial selective pressure. The *E*. *aerogenes* G7 chromosome also contains four intact prophages, which differ from the genome of *E*. *aerogenes* KCTC2190, four IS elements, and one ICE (Table 3, S2 Fig, S1 and S2 Datasets). Multiple genome alignments obtained by progressiveMauve were used to compare the overall genome architecture of EAG7 to that of other strains—including the genomes of the other isolates and the previously published genomes of strains KCTC2190 and EA1509E [29, 44]. Some of these alignments are shown supplementary information (S3 Fig). Overall, the *E*. *aerogenes* isolates appeared to be characterized by a low degree of divergence and the genetic synteny among the chromosomes was conserved. The alignment of EAG7 to EA1509E shows the existence of fourteen locally collinear blocks (LCBs) of witch twelve correspond to the chromosome and to the large plasmid pGPN1 (Figure A in S3 Fig). This analysis also indicates that the isolates can be separated into distinct populations. Strains G2 to G7 have syntenic genomes with highly conserved sequences and only one putative rearrangement located on pGPN1 (Figure C in S3 Fig: inversion of LCBs 2 and 3 relative to G7 genome). On the opposite, the genome of G1 exhibits more rearrangements and modifications when compared to that of G7, thus indicating that G1 is more distant to G7 than the other G isolates (Figure B in S3 Fig). These modifications include the absence of three regions of 42–48 Kbp associated with phage functions and nearly 103 Kbp of additional sequences (S2 Fig, S1 and S2 Datasets). Strains P2 to P5 also present genome rearrangements and modifications compared to G7 (Figure E in S3 Fig). Among these, one prophage region of 48 Kbp was absent, around 98 Kbp of supplementary sequences, corresponding to the Imp locus (involved in type VI secretion) and phage functions, and a supplementary plasmid of about 35 Kbp, similar to pN-Cit from *C*. *freundii* strain CFSTE (S2 Dataset) were found. ProgressiveMauve plots also show that isolate P1 is clearly distant from all the other isolates but shares a good synteny with the reference strain KCTC2190 (Figure A in S3 Fig). For this reason, isolate P1 was not further studied.

**Table 3:** Features of the G7 genome. CDS, coding sequences; ICE, integrative and conjugative element; IS, insertion sequence. * Data refer to the chromosome only, except when plasmids are indicated.

	E. aerogenes G7 *
Size (bp)	5,452,368
GC content (%)	54.92
CDS (n)	5,267
rRNAs (n)	
16S	8
23S	8
5S	9
tRNAs (n)	87
Plasmids (n)	2
Prophages (n)	8
PhageG7.1	Intact
PhageG7.2	Intact
PhageG7.3	Questionable
PhageG7.4	Intact
PhageG7.5	Incomplete
PhageG7.6	Questionable
PhageG7.7	Intact
PhageG7.8	Questionable
ICEs (n)	1
IS elements (n)	4
IS5075	3
IS1351	1

### Comparative genomics between the clinical isolates and identification of mutations associated to MDR

Comparative genome sequencing of all twelve isolates pointed to several mutations (see S2 Dataset for a complete list of SNPs and InDels). Total, we found 70–72 and 9–11 mutations in the genome of isolates P2-P5 and G2-G6, as compared to that of G7, respectively. These values confirm that the isolates are closely related. Interestingly, only a few numbers of non-synonymous mutations were specifically present in the core genome of MDR isolates. The QRDRs in all twelve isolates, including *gyrA*, *gyrB*, *parC* and *parE* genes, contained several amino acid substitutions (Ser67Ala and Thr83Ile in GyrA and Ser80Ile in ParC for isolates P2-P5 and G1; Thr83Ile and Asp87Asn in GyrA and Ser80Ile in ParC for isolates G2-G7), when sequences were compared to that of KCTC2190. These mutations likely explain quinolone resistance in theses isolates (Table 1 and S1 Table). Additional mutations mapped to 6 *loci* that are known to control outer membrane permeability. Frameshift mutations introducing stop codons in the structural gene of Omp36 were systematically identified in all MDR isolates—P2, P4, P5, G2, G5 and G6. Isolate G2 also carried a mutation in the structural gene of Omp35. Isolates P4-P5 and G5 contained non-synonymous mutations in regulatory genes: P4 and P5 were mutated in *cpxA* (generating a Tyr144Asn substitution); an intergenic mutation between *cpxP* and *cpxRA* was found in P5; G5 was mutated in *rob* (generating a Gly140Ser substitution), *phoQ* (generating a Leu348Gln substitution) and *pmrB* (generating a Thr157Pro substitution). Rob is a global transcription factor of the AraC/XylS family. MarA, SoxS and Rob have been shown to function in co-operative manner to induce MDR in response to a variety of external stresses [45, 46]. CpxA, PhoQ and PmrB (BasS) are sensor kinases of the CpxRA, PhoPQ and PmrB (BasRS) two-component regulatory systems, respectively. The CpxRA two-component system has been extensively studied and reported as a major pathway for outer membrane stress response [47]. Notably, activation of CpxRA down regulates the expression of outer membrane proteins [48]. On the other hand, PhoPQ and PmrAB two-component systems control the expression of LPS-modifying enzymes [49, 50]. These data now offer a unique opportunity to study the influence of two-component regulatory systems on outer membrane permeability, including porin expression and LPS modification, and MDR phenotypes in clinical isolates. Nonetheless, the observation that these mutations appeared during imipenem chemotherapy in parallel with the emergence of carbapenem-resistance strongly suggests that they provide an adaptive response to the antibiotic stress.

## Discussion

**Fig 5:**
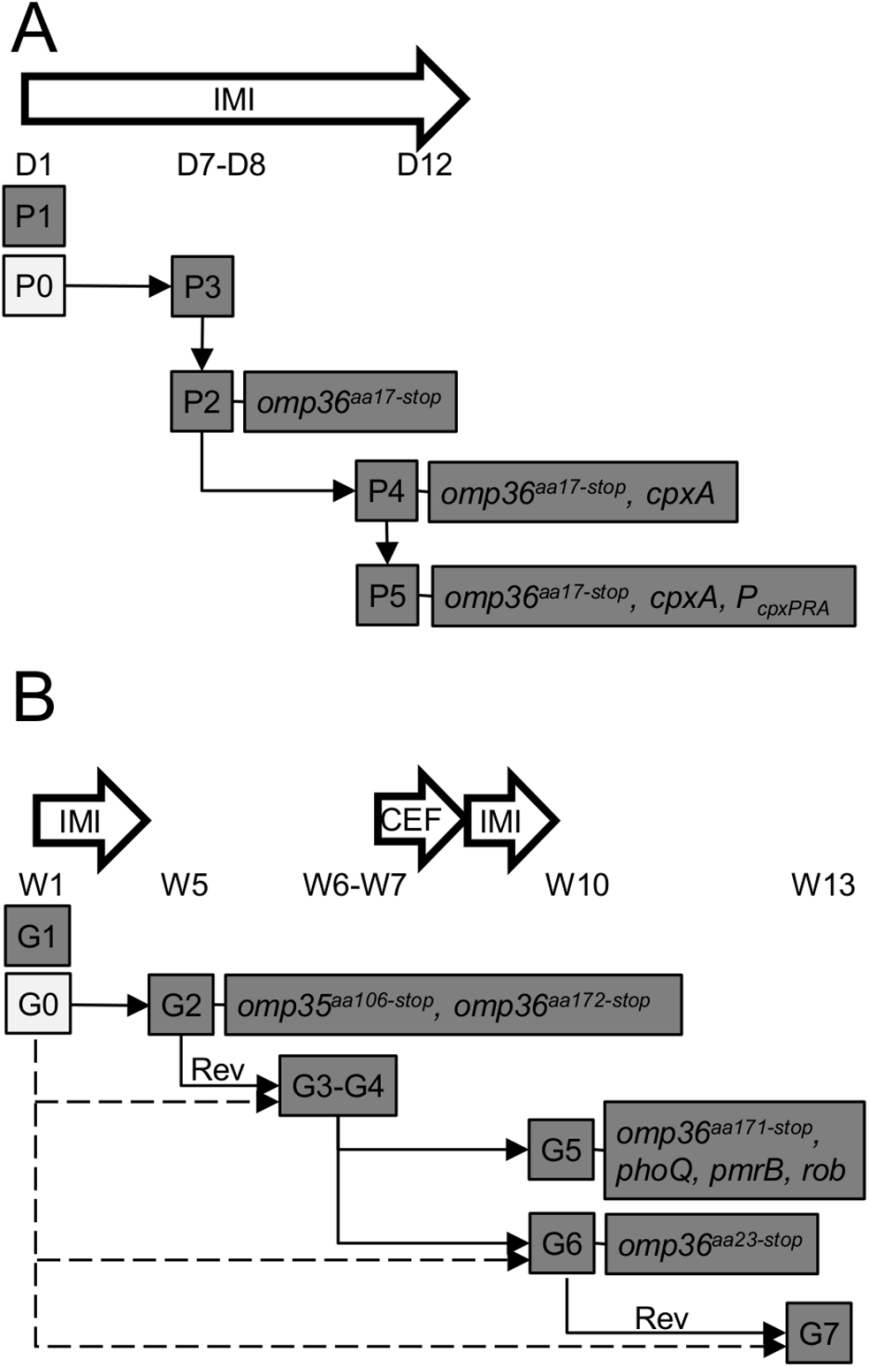
Model of intra-patient evolution of *E*. *aerogenes* during antibiotic treatment. Plain arrows represent the putative evolution of bacterial subpopulations in patient P (A) and patient G (B). The genomic distance between the first isolates (P1 and G1) and the subsequent strains (P2-P5 and G2-G7) indicates that these have evolved from other ancestors (P0 and G0). Lack of evidence of genotypic reversion (Rev) from resistant to susceptible strains within the G series suggest alternative evolutionary pathways represented by dotted arrows. Clinical timelines are indicated from day (D) 1 to D12 and week (W) 1 to W13 for patient P and patient G, respectively. IMI, imipenem; CEF, cefpirome.

Carbapenem-resistant *Enterobacteriaceae* are considered as an urgent threat worldwide. A first step to combat this threat is to understand the genomic traits of resistant clinical isolates as a mean to get new insight into their successful adaptation as nosocomial pathogens. It is also important to consider the genome evolution of clinical strains isolated from patients at timed intervals during antibiotic treatments. Herein, we performed whole genome sequencing (WGS) on a total of twelve clinical strains of *E*. *aerogenes* isolated from two patients treated with imipenem and showing variable MDR phenotypes. We have thoroughly mined the genome of the imipenem-susceptible EAG7. Of note, as EA1509E and EAG7 both belong to a same epidemic clone, we now have access to two completely determined and closed genomes of *E*. *aerogenes* representatives involved in large hospital outbreaks in France. The genome of EAG7 is composed of one chromosome and two plasmids of different sizes. It contains multiple resistance markers, including β-lactamases and efflux pumps, which can contribute to the successful adaptation of this strain to the hospital environment. WGS of the twelve isolates followed by comparative genomics using EAG7 as a reference allowed us to determine the genome evolution of the strains in the patient host and identify putative mutations responsible for MDR (S2 and S3 Datasets). For patient P, isolate P1, which was found closely related to KCTC2190, disappeared rapidly after the first use of imipenem. Analysis of SNPs in the P2-P5 isolates rather suggests the co-existence of an imipenem-susceptible ancestor resembling P3. Then, constant exposure to antibiotic stress during the whole period of treatment fixed mutations that occurred in a stepwise manner in this population in strict correlation with the acquisition of new phenotypic resistances (Fig 5A). Likewise, isolate G1 did not evolve during antibiotic treatment, suggesting the co-existence of an imipenem-susceptible ancestor that subsequently yielded G2-G7. However, one cannot rule out the possibility of an exogenous infection. For patient G, discontinuity of the treatment lead to several genotypes associated to MDR as well as genotypic-phenotypic reversions (Fig 5B). Notably, periods of imipenem treatment induced porin-loss concomitant to carbapenem-resistance.

In this study, we used different assays to analyze and correlate the phenotypic and genotypic characteristics of two series of *E*. *aerogenes* clinical isolates. We found no mutation in the *mar*, *ram* or *sox* region that could differentiate G7 from the other isolates. Only isolate G5 has a single base pair exchange in *rob* that generates the amino acid substitution Gly140Ser in Rob. Previous results from an heterogeneous collection of our clinical isolates showed the presence of multiple but conserved mutations in both *ramA* and *ramR*, when sequences were compared to that of the reference strains ATCC15038 and ATCC13048 (A. Molitor, A.V. Davin-Régli and J. M. Pagès, unpublished data). Isolates G6 and P2 were part of this collection. All had a 5 bp deletion upstream of *ramA*, between the marbox and the translational start; 3 bp exchanges that generated 3 amino acid substitutions (Ala72Asp, Pro100Ser and Ile121Ser) and a frameshift mutation that altered and shortened the C-terminus of RamR. Interestingly, q-PCR analysis also showed these three isolates had increased levels of *ramA* and *acrA* transcripts, together with decreased levels of *omp35* transcripts, in comparison to the reference strains. However, the impact of the mutated *ramA* or *ramR* on MDR and efflux pumps expression has not been tested.

Our data suggest that both reduced outer membrane permeability and expression of β-lactamases, but not efflux, are the main contributors of MDR in these isolates. Indeed, a previous study showed that all twelve isolates belong to a prevalent epidemiologic clone that contains a plasmid encoding a TEM-24 ESBL [7]. Moreover, the presence of an inducible chromosomal AmpC β-lactamase has been detected in the isolates, with the exception of G1 (data not shown). These data were confirmed by WGS and likely explain the high resistance levels to cephalosporins even in the presence of a β-lactamase inhibitor (i.e. TEM-24 but not AmpC is inhibited by clavulanic acid) (S1 Table). Here, it is also worth to note that the *bla*_*TEM-24*_ gene located on pGPN1 is flanked sequences of IS*Swi1*, thus could be subject to duplication by recombination between regions of homology (S1 Dataset). Gene duplications are relevant to the evolution of resistance in that these events are frequent and instable.

High resistance levels to quinolones are generally due to increased efflux activity and/or target mutations. Here, active efflux has been detected in all the isolates using fluorimetric assays (Figs 2 and 3). However, the use efflux inhibitors such as PAβN or NMP did not restore susceptibility to quinolones but only that of erythromycin (S1 Table). This strongly suggests the presence of mutations in QRDRs of DNA gyrase and/or topoisomerase IV subunits.

Although all the isolates produce β-lactamases, resistance to carbapenems is exclusively related to the loss of Omp36. Consistently, susceptibility to β-lactams can only be restored by the addition of PMBN, a derivative of polymyxin B that increases outer membrane permeability and facilitates antibiotic permeation (S1 Table). The absence or the decreased expression of the two major porins (OmpF and OmpC in *E*. *coli*, Omp35 and Omp36 in *E*. *aerogenes* or OmpK35 and OmpK36 in *K*. *pneumoniae*) in combination with the presence of β-lactamases has been previously implicated in carbapenem-resistance [4, 6–10, 23, 51]. Here, we found that none of the clinical isolates expressed Omp35 while Omp36 was only expressed in the carbapenem-susceptible strains. WGS data were used to investigate the basis of porin loss. In all cases, the loss of Omp36 resulted from frameshift mutations in the coding sequence leading to a premature translational stop. In contrast, the loss of Omp35 is less clear, with the exception of isolate G2 in which *omp35* is inactivated by a frameshift mutation (S2 and S3 Datasets). We suspect molecular mechanisms working at the transcriptional or post-transcriptional level or preventing porin insertion into the outer membrane. Part of this question will be addressed by transcriptomic analysis.

Special attention will also be given to missense mutations that were identified in three different sensor kinase two-component regulatory systems—*cpxA* in isolates P4-P5, and *phoQ* and *pmrB* in isolate G5. Such mutations are not uncommon in bacteria and have been linked to antimicrobial resistance [52–58]. Mutations in the *cpxA* have been shown to contribute to amikacin and cephalosporin resistance in *E*. *coli* and *Salmonella enterica* serovar Typhimurium, respectively [52]. Mutations in *phoQ* and *pmrB* have been identified in multiple pathogens including *S*. *enterica*, *P*. *aeruginosa* and *K*. *pneumoniae* [53–58]. These two-component systems control the expression of genes linked to LPS modification and promote resistance to polycationic antimicrobials, including polymyxins and antimicrobial peptides. Consistently, we observed that isolate G5 shows high resistance level to polymyxin B (S1 Table). Future works will aim to study the role of two-component systems in antibiotic resistance and examine these systems as potential targets for the development of drugs to potentate uptake and activity of existing antibiotics.

## Supporting Information

S1 Dataset RNA, ICE, IS and prophages. (RAR)

S2 Dataset SNPs, InDels and regions of divergence. (RAR)

S3 Dataset Antibiotic resistance markers. (RAR)

S1 Fig SDS-PAGE of membrane proteins. Porin-plus isolates (G3, G4, G7, P1 and P3) were cultured overnight in NB. Strain ATCC15038 was grown in NB (in which both Omp35 and Omp36 are expressed) and NBS (in which the expressed of Omp35 is severely repressed), and used as a control. Membrane fractions were prepared as described in the Materials and Methods section. Membrane proteins were separated by Urea-SDS-PAGE and stained with Coomassie Blue. Bands of interest (b1-b8) were excised, digested with trypsin, and submitted to mass spectrometry Nano-LC MS/MS analysis. Peptides were mapped on the amino acid sequence of Omp35 and Omp36 deduced from the genome sequence of isolate G7 (S2 Table).
(DOCX)

S2 Fig Analysis of prophages. This figure was generated by PHAST (http://phast.wis/hartlab.com)
(DOCX)

S3 Fig Examples of Mauve plots of *E*. *aerogenes* sequenced genomes. A shows genome alignments of EAG7, EA1509 [44] and KTCTC2190 [29]. B-F show genome alignments of G7 and G1, G7 and G1, G7 and P1, G7 and P2, and P1 and KCTC2190, respectively. Boxes with identical colors represent local collinear blocks (LCBs), indicating homologous DNA regions shared by two or more chromosomes without sequence rearrangements. LCBs indicated below the horizontal black line represent reverse complements of the reference LCB. White segments within LCBs represent strain-specific regions. Red bars delineate contigs.
(DOCX)

S1 Table Minimum inhibitory concentrations (MIC). MIC values are expressed in μg/ml.
(XLSX)

S2 Table Identification of porin by mass spectrometry. (DOCX)

S3 Table Quantification of H33342 influx and 1,2’ DNA efflux. Quantitative values of H33342 influx (^*a*^) and DNA efflux (^*b*^) are derived from graphs shown in Fig 1 and Fig 3, respectively. *m* are initial slopes measured as changes in fluorescence intensities (FI) per second.
(DOCX)
