# Supplementary figures and images for: In Vivo Evolution of Bacterial Resistance in Two Cases of Enterobacter aerogenes Infections during Treatment with Imipenem
Source: PLoS One. 2015 Sep 23;10(9):e0138828. doi: 10.1371/journal.pone.0138828 (PMC4580588; doi:10.1371/journal.pone.0138828)

**S2 Fig**


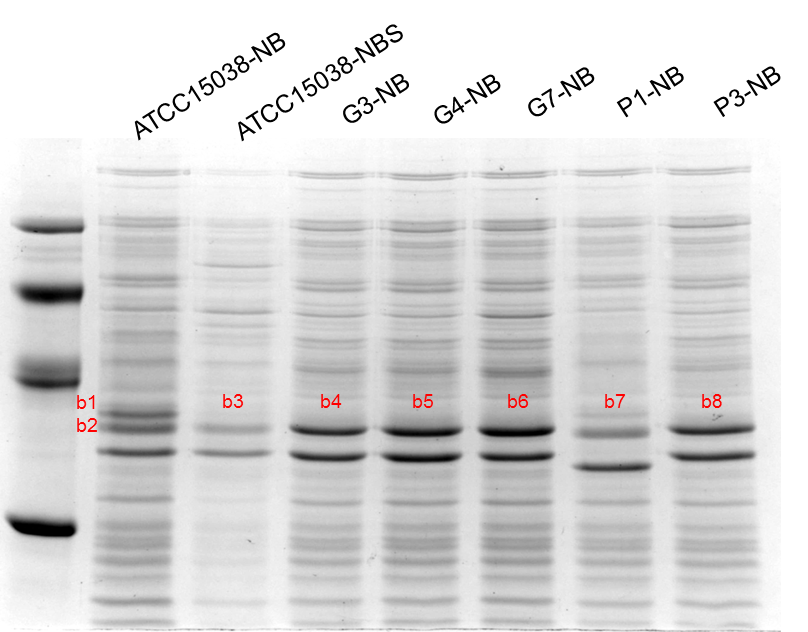

Supplement: S1 Fig — Porin-plus isolates (G3, G4, G7, P1 and P3) were cultured overnight in NB. Strain ATCC15038 was grown in NB (in which both Omp35 and Omp36 are expressed) and NBS (in which the expressed of Omp35 is severely repressed), and used as a control. Membrane fractions were prepared as described in the Materials and Methods section. Membrane proteins were separated by Urea-SDS-PAGE and stained with Coomassie Blue. Bands of interest (b1-b8) were excised, digested with trypsin, and submitted to mass spectrometry Nano-LC MS/MS analysis. Peptides were mapped on the amino acid sequence of Omp35 and Omp36 deduced from the genome sequence of isolate G7 (S2 Table). (DOCX) [file pone.0138828.s004.docx]

**S5 Fig**


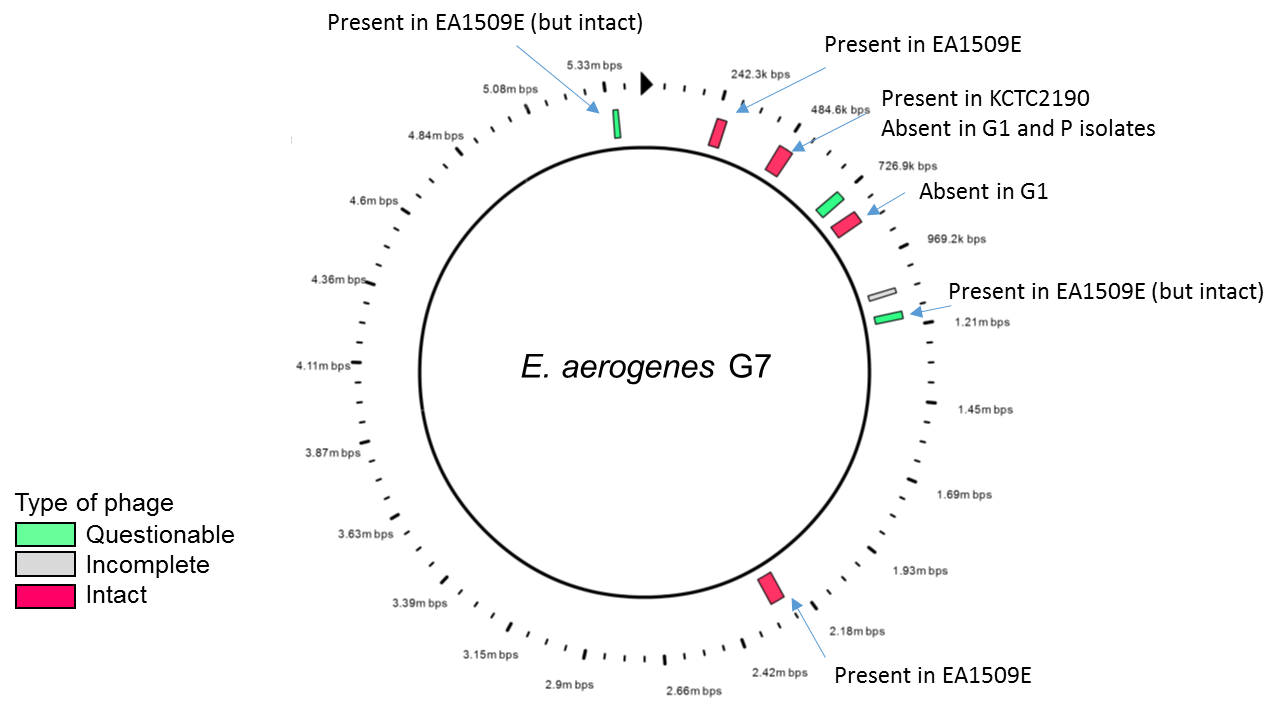

Supplement: S2 Fig — This figure was generated by PHAST (http://phast.wis/hartlab.com) (DOCX) [file pone.0138828.s005.docx]

**S6 Fig**


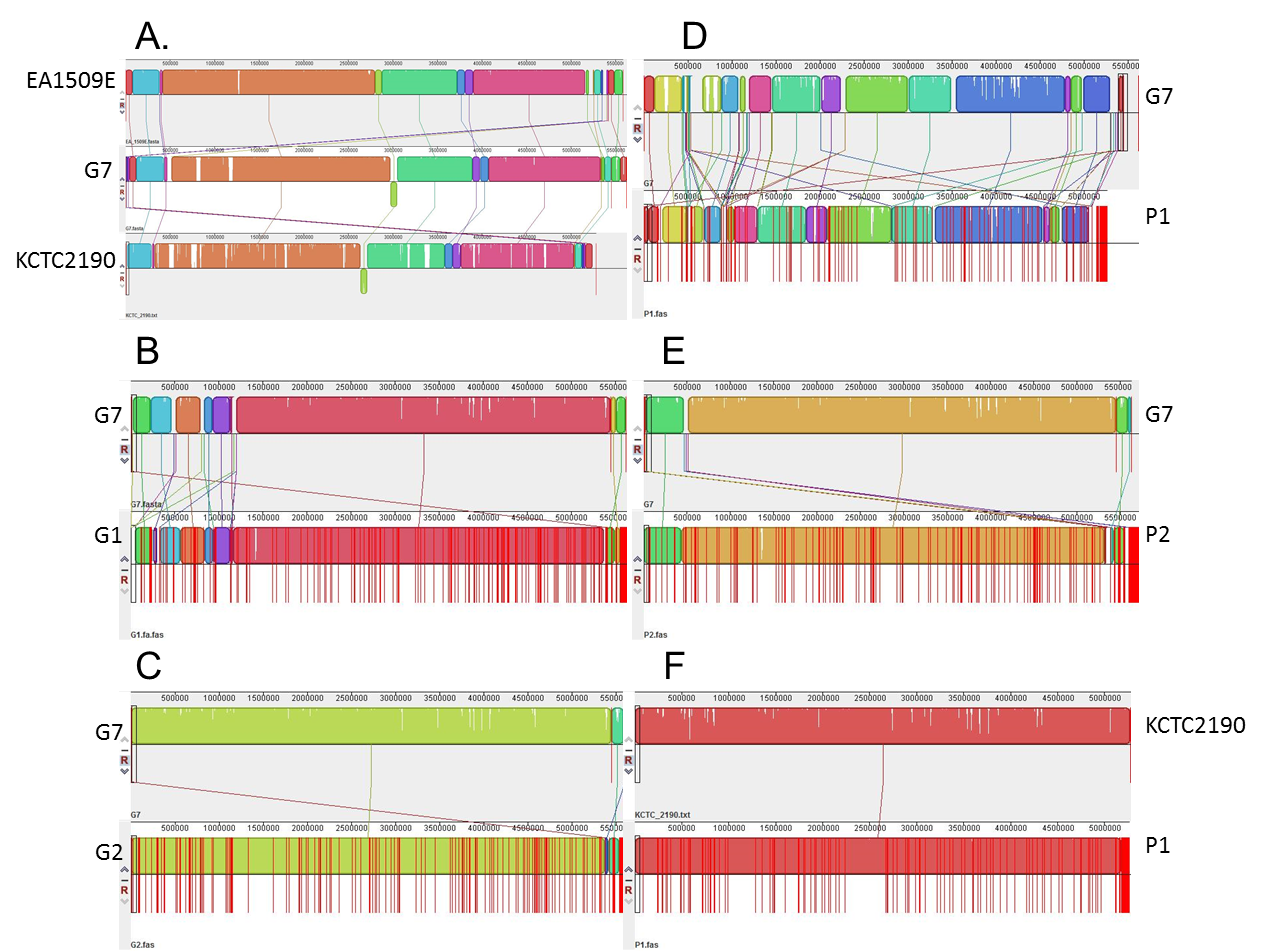

Supplement: S3 Fig — A shows genome alignments of EAG7, EA1509 [44] and KTCTC2190 [29]. B-F show genome alignments of G7 and G1, G7 and G1, G7 and P1, G7 and P2, and P1 and KCTC2190, respectively. Boxes with identical colors represent local collinear blocks (LCBs), indicating homologous DNA regions shared by two or more chromosomes without sequence rearrangements. LCBs indicated below the horizontal black line represent reverse complements of the reference LCB. White segments within LCBs represent strain-specific regions. Red bars delineate contigs. (DOCX) [file pone.0138828.s006.docx]
