# Supplementary material for: In Vivo Evolution of Bacterial Resistance in Two Cases of Enterobacter aerogenes Infections during Treatment with Imipenem
Source: PLoS One. 2015 Sep 23;10(9):e0138828. doi: 10.1371/journal.pone.0138828 (PMC4580588; doi:10.1371/journal.pone.0138828)
Supplement: S2 Table — (DOCX) [file pone.0138828.s008.docx]

**S3 Table**

| Sample | Identification | Sequence coverage (%) | Peptides (n) |
| --- | --- | --- | --- |
| b1 | Omp35 | 41 | 11 |
| b2 | Omp36 | 29 | 12 |
| b3 | Omp36 | 26 | 12 |
| b4 | Omp36 | 21 | 12 |
| b5 | Omp36 | 32 | 13 |
| b6 | Omp36 | 41 | 17 |
| b7 | Omp36 | 27 | 12 |
| b8 | Omp36 | 41 | 15 |
