# Supplementary material for: In Vivo Evolution of Bacterial Resistance in Two Cases of Enterobacter aerogenes Infections during Treatment with Imipenem
Source: PLoS One. 2015 Sep 23;10(9):e0138828. doi: 10.1371/journal.pone.0138828 (PMC4580588; doi:10.1371/journal.pone.0138828)
Supplement: S3 Table — Quantitative values of H33342 influx (a) and DNA efflux (b) are derived from graphs shown in Fig 1 and Fig 3, respectively. m are initial slopes measured as changes in fluorescence intensities (FI) per second. (DOCX) [file pone.0138828.s009.docx]

**S4 Table.**

| Strain | *m_influx_* (ΔFI/s)*^a^* | *m_efflux_* (ΔFI/s)*^b^* |
| --- | --- | --- |
| ATCC 15038 | 30,9 | -5,2 |
| G1 | 33,5 | -6,4 |
| G2 | 36,4 | -2,8 |
| G3 | 31,4 | -2,5 |
| G4 | 26,5 | -2,1 |
| G5 | 34,4 | -1,4 |
| G6 | 30,8 | -2,0 |
| G7 | 32,9 | -3,2 |
| P1 | 30,4 | -9,2 |
| P2 | 29,2 | -10,7 |
| P3 | 28,8 | -12,2 |
| P4 | 27,3 | -11,1 |
| P5 | 27,4 | -11,2 |
